# Engaging Community Stakeholders to Evaluate the Design, Usability, and Acceptability of a Chronic Obstructive Pulmonary Disease Social Media Resource Center

**DOI:** 10.2196/resprot.3959

**Published:** 2015-01-28

**Authors:** Michael Stellefson, Beth Chaney, Don Chaney, Samantha Paige, Caroline Payne-Purvis, Bethany Tennant, Kim Walsh-Childers, PS Sriram, Julia Alber

**Affiliations:** ^1^Center for Digital Health and WellnessDepartment of Health Education and BehaviorUniversity of FloridaGainesville, FLUnited States; ^2^Department of Health Education & PromotionEast Carolina UniversityGreenville, NCUnited States; ^3^Department of Health and KinesiologyMississippi University for WomenColumbus, MSUnited States; ^4^ICF InternationalFairfax, VAUnited States; ^5^Department of JournalismUniversity of FloridaGainesville, FLUnited States; ^6^Department of MedicineDivision of Pulmonary, Critical Care, and Sleep MedicineUniversity of FloridaGainesville, FLUnited States

**Keywords:** COPD, health communication, social media, patient education

## Abstract

**Background:**

Patients with chronic obstructive pulmonary disease (COPD) often report inadequate access to comprehensive patient education resources.

**Objective:**

The purpose of this study was to incorporate community-engagement principles within a mixed-method research design to evaluate the usability and acceptability of a self-tailored social media resource center for medically underserved patients with COPD.

**Methods:**

A multiphase sequential design (qual → QUANT → quant + QUAL) was incorporated into the current study, whereby a small-scale qualitative (qual) study informed the design of a social media website prototype that was tested with patients during a computer-based usability study (QUANT). To identify usability violations and determine whether or not patients found the website prototype acceptable for use, each patient was asked to complete an 18-item website usability and acceptability questionnaire, as well as a retrospective, in-depth, semistructured interview (quant + QUAL).

**Results:**

The majority of medically underserved patients with COPD (n=8, mean 56 years, SD 7) found the social media website prototype to be easy to navigate and relevant to their self-management information needs. Mean responses on the 18-item website usability and acceptability questionnaire were very high on a scale of 1 (strongly disagree) to 5 (strongly agree) (mean 4.72, SD 0.33). However, the majority of patients identified several usability violations related to the prototype’s information design, interactive capabilities, and navigational structure. Specifically, 6 out of 8 (75%) patients struggled to create a log-in account to access the prototype, and 7 out of 8 patients (88%) experienced difficulty posting and replying to comments on an interactive discussion forum.

**Conclusions:**

Patient perceptions of most social media website prototype features (eg, clickable picture-based screenshots of videos, comment tools) were largely positive. Mixed-method stakeholder feedback was used to make design recommendations, categorize usability violations, and prioritize potential solutions for improving the usability of a social media resource center for COPD patient education.

## Introduction

Approximately 12.7 million adults have chronic obstructive pulmonary disease (COPD) [1] and experience complications, such as breathing exacerbations that require frequent hospitalization [2]. Patients with COPD generally receive little information on the social and behavioral dimensions of living with breathing problems, such as techniques to improve self-management, self-efficacy, and self-regulation of dyspnea (ie, shortness of breath) [3,4]. Moreover, few patients with COPD are ever referred to pulmonary rehabilitation, primarily because most programs operate in outpatient hospital settings [5,6]. Pulmonary rehabilitation helps provide patient education on rehabilitative skills such as pursed-lipped and diaphragmatic breathing, stress management, and customized exercise [7,8]. With limited instruction and skill-building resources available to patients, a majority of patients living with COPD are unable to modify their lifestyles, which ultimately increases their risk of hospitalization [9]. Research suggests the most significant improvements in reducing health care utilization due to COPD complications have been achieved from educational interventions designed to meet the dynamic self-management learning needs of patients [3,10-12].

Educational programs in COPD management interventions frequently include smoking cessation, medication use, exercise, breathing strategies, exacerbation prevention, and stress management [13]. Patient education can help individuals living with COPD to achieve fundamental objectives such as increased knowledge and self-efficacy, which are both associated with exacerbation-related health care utilization [10,14,15]. While patient education is fundamental to improving outcomes in COPD, older patients often experience impairments to memory and abstract reasoning which causes low health literacy [16,17], limited compliance with complex self-management guidelines [18], and increased dependence on the health care system [19,20]. Low health literacy also often goes unrecognized by health care providers [11,18].

Over the past decade, the Internet has become a common place where patients with chronic disease can access health information, improve health literacy, and interact socially with peers regarding common health conditions [21-24]. Patients with COPD have participated in online self-management programs that have shown moderate levels of usability and effectiveness [25,26]. However, one previous usability study of an eHealth behavior change intervention in COPD asked participants to read lengthy tailored messages in fixed educational modules, which was ultimately determined to be too much health information for most patients to process [26]. Patients with chronic disease generally report difficulty accessing comprehensible disease-related content on the Internet, which has led to slow adoption of consumer health care technologies for chronic disease self-management [27-29]. In particular, elderly and minority patients commonly experience difficulty using two-way health communication technologies (Web 2.0) [24,30], which often results in greater attrition and nonuse of online, chronic disease self-management interventions [31,32]. Therefore, the design of easy-to-use, interactive websites for patients living with chronic disease is becoming increasingly important as the shift towards patient empowerment and self-control of health outcomes continues to become more and more pervasive throughout the health care system [33,34].

Information and communication technologies, such as social media, have the potential to expand the reach of strategic health communication interventions that promote disease prevention and life-saving health protective behaviors. Social media is a “tool or platform that derives its content and principal value from user engagement and permits those users to interact with content as part of a larger movement in communications organized under Web 2.0” [35]. Specifically, social media is well suited for providing patients with motivational messages and key behavioral change resources that prompt and facilitate good health [34]. Creating easy-to-use social media resources that enable two-way health communication among patients with chronic disease(s) and their informal caregivers (eg, friends, family members) may help to extend the efficacy of traditional patient education on self-management [32,36].

Older adults with chronic disease often possess low eHealth literacy, or a low ability to seek, find, understand, and appraise online health information and apply knowledge gained to addressing or solving a health problem [37]. For these reasons, it is important to explore the use of empowering, low-tech self-management tools that can be effectively controlled by the user. To prevent user dissatisfaction and abandonment of technology, consumer health informatics tools should be designed to allow even the novice user to interact with, and manipulate, the interface to accomplish personal task objectives with few errors [38]. Self-tailored, chronic disease self-management programs structure interactive, self-directed learning experiences that cover topics such as stress management, personal fitness/exercise regimens, and preparing healthy meals. The concept of self-tailoring patient education is based on self-efficacy theory [39]. This theory proposes that sociocognitive support resources are best suited to help groups and individuals pursue mastery experiences that build self-confidence for initiating, performing, and maintaining time-bound behavioral action plans that are both beneficial and achievable [40]. Vicarious learning opportunities facilitated through interactive health communication technology represents a practical approach to using Web 2.0 for self-management that can augment patient-provider communication and motivate health behavior change. A systematic review of Web 2.0 chronic disease self-management interventions revealed that these types of programs may reduce health distress and activity limitation, improve health status, and foster more active patient engagement [32].

To date, no usability studies have evaluated patient use of popular social media to access and engage in online COPD patient education on respiratory therapy and self-management support. Usability testing is a fundamental step in patient-centered technology design that uses a systematic process to evaluate end users’ goals such as efficiency, avoiding errors, satisfaction, and learnability [33]. Usability studies enable researchers to discover strengths and weaknesses in prototype technologies by exploring end users’ experiences using new technologies in controlled computer laboratory settings [41]. In contrast, community-engaged research (CEnR) methods take place outside of controlled laboratory settings. These methods are designed to foster collaborations with, and among groups of, people affiliated by geographic location, special interest, or similar situations with the goal of addressing issues that affect the health and well-being of the group [42]. Engaging the community of interest during the development and testing of consumer health technologies is critical to ensuring that potential users feel a part of the development process and find the product to be applicable and practical for use [42]. Therefore, the purpose of this study was to use community-engaged research principles within a mixed-method research design to evaluate a self-tailored, social media website prototype for medically underserved patients with COPD.

To maximize benefits for medically underserved patients, our focus was on stimulating users without overburdening them with lengthy text, excess content, and potentially disorienting technical features (eg, pop-ups). Clickable screen captures of 167 discrete respiratory therapy and COPD patient education video segments were uploaded into a structured social media platform. Formative testing and validation of this video content is reported elsewhere [36,43,44]. Literacy-sensitive comment boxes were attached to each video with built-in breaks for visual clarity. A heuristic evaluation of the preliminary technical architecture version of the social media website prototype was conducted by a small panel of external health care communication experts [38]. Results from this evaluation helped to identify usability violations in the initial website prototype design prior to undertaking the current study. This involved conducting a community-engaged usability and acceptability evaluation with medically underserved patients with COPD.

## Methods

### Procedure

#### Overview

An adapted version of the Website Development Model for the Healthcare Consumer (WDMHC) [45] was used to evaluate the user-centered social media website prototype built according to user characteristics (eg, age, gender, education, race/ethnicity, health status, eHealth literacy) and information goals supported through appropriate technical functions. Specifically, three phases of the WDMHC that were used were (1) evaluation of technological design and features using interviews with potential users and experts, (2) computer-based usability testing using think-aloud methodology, and (3) one-on-one retrospective interviews with patients using validated interview rubrics and questionnaires.

The Priority-Sequence Model [46] was used to assist in the priority and sequence decision making for the usability research design. A multiphase sequential design (qual → QUANT → quant + QUAL) [47] was incorporated into the current study, whereby a small-scale qualitative (qual) study informed the design of a social media website prototype that was evaluated during a primary computer-based usability study (QUANT). Results from the computer-based usability study were explored via validated questionnaires (quant) and retrospective, in-depth, semistructured interviews (QUAL), where patients were given the opportunity to discuss usability strengths and weaknesses present within the social media website prototype following their computer-based sessions. Moreover, what was learned during the sequential phases of developmental research helped the research team to identify high-priority technical and formatting modifications for the COPD social media resource center. The study protocol was approved by the University of Florida’s Institutional Review Board (IRB)-01, within its medical center.

#### Phase I: Evaluation of Design and Features of Social Media Website Prototype (qual)

##### Sample

In this study, “community” was operationally defined as stakeholders affected by, or involved with, the treatment of COPD. Community stakeholders included patients with COPD, informal caregivers (eg, friends, family members) of those with COPD, clinicians who treat patients with COPD, researchers who study COPD, community health agencies that advocate on behalf of patients with COPD, and any other individuals or organizations involved with providing health services to patients with COPD. Therefore, 5 medically underserved patients with COPD and 5 health care communication experts, who possessed experience working with patients living with COPD, were interviewed to gather information on desirable features of a mock-up for a COPD patient education social media website. Patient interviewees were recruited by research navigators affiliated with a CEnR program at a large research-intensive university in the southeastern United States. This particular CEnR program has a mission to eliminate research disparities based on access, race, and age, by fostering communication and reciprocal trust between traditionally underrepresented communities and academic researchers. The health care communication experts interviewed in this study included an internationally renowned patient education researcher, an experienced health communication researcher with experience designing Web-based applications for underserved populations, an applied physiologist with clinical experience directing hospital-based pulmonary rehabilitation programs, an expert on using exercise therapy in older adult populations, and a medical doctor with specialty in pulmonary medicine.

##### Procedures

**Figure 1:**
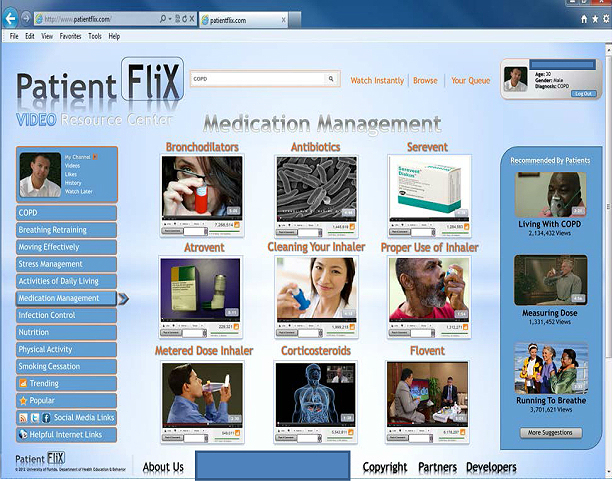
Original mock-up of the online COPD patient education social media website.

All patient and expert interviewees were sent a brief description of the site’s intended purpose via email, and were asked to review a mock user interface of the social media website prototype (Figure 1).

The preliminary social media website prototype design was conceived to follow specific user-centered design principles appropriate for older adults [48]. The website membership model accommodated all authenticated users with a single sign-on process using unique log-in credentials (ie, usernames and passwords). All text files, documents, and images were integrated into the prototype and converted using Adobe Dreamweaver, a Web-development application, and uploaded using HTML files as mock-up screens. To maximize the appeal of the social media website prototype among potentially nontech-savvy patients with COPD, we used concrete and realistic visuals with clear captions. We also adopted several design recommendations suggested by Choi and Bakken [33], who developed a Web-based educational portal for parents with children in neonatal intensive care units. These adopted recommendations included (1) using an ordered format for topics, (2) listing categories clearly, (3) keeping pages short and concise, (4) using large buttons adequately spaced apart, and (5) maintaining design simplicity in drop-down menus and window placement. The simple design and shallow Web architecture of the social media website prototype benefitted from a contrasting color scheme, consistent sans serif fonts, minimal amount of written text, and one-touch point-and-click access to most site applications. All website functions and navigation tools were outlined using purposeful graphical representations and information displays that complied with our content strategy and visual design standards for medical information sites on the Internet [48-50]. Results from the patient and expert interviews helped the researchers create a functional social media website prototype.

#### Phase II: Computer-Based Usability Testing With Patients (QUANT)

##### Sample

Typically, 5 to 12 individuals representing the intended user audience are involved in think-aloud testing [49,51]. In this study, a convenience sample of 8 English-speaking adults with COPD was referred into the study by study navigators at the university’s CEnR program. Individuals were eligible to participate if they met the following inclusion criteria: (1) registered in the CEnR program, (2) willing and able to travel to the community health center, (3) 40 years of age or older at the time of enrollment, (4) able to speak and read the English language, and (5) possessed at least some experience using the Internet over the previous 3 months. Participants were excluded from participating if they (1) had a history of major cognitive impairments or comorbid psychiatric illnesses which could adversely impact their ability to understand and use a website, and/or (2) were interviewed during Phase I of the study. All potential participants were made aware that they would receive a US $40 gasoline/supermarket gift card upon completing the session.

##### Think-Aloud Protocol

The think-aloud usability method [51] measures performance on typical tasks for which a particular end-user technology is designed. It primarily consists of two stages: (1) eliciting and recording end users’ cognitive thoughts while they attempt to navigate and complete tasks using the technology, and (2) measuring and analyzing the recorded thoughts and interactions of end users using the technology by following a standard protocol [52]. The goal of the computer-based think-aloud testing was to provide an in-depth understanding of each patient’s experience of the following: (1) navigating the social media website prototype, (2) finding and commenting on posted videos, and (3) using interactive comment boxes with threaded discussion forums. These sessions took place at the university’s community health center in a controlled computer laboratory, where all participants used the same laptop computer. Sessions lasted approximately one hour. The protocol was pilot-tested with one COPD patient who was not involved with participating in the actual usability testing.

Prior to beginning each usability session, one member of the research team provided each patient with a brief explanation of the research study, including information on human subject protections, audio/video recording, anonymity, and confidentiality of the data to be collected. It was emphasized to each patient that the study was not designed to evaluate the user’s ability to use the website, but rather it was a test of whether the website worked as intended (ie, test of the website, not the user). Following informed consent, the moderator, who was not involved in the development of the social media website prototype, provided brief instructions to each patient regarding how to use the laptop keyboard and external mouse. Following this brief training, patients were instructed to familiarize themselves with the social media website prototype layout and develop a unique log-in (username and password). Each registered patient was then asked to complete a representative sample of nine social media tasks (Table 1). To account for variability in user search preferences and operational skills [53,54], the think-aloud protocol included both directed (ie, asking for specific information) and semidirected (ie, open ended to allow for multiple solutions) social media tasks. For example, patients were asked to search for particular video titles and screenshots (directed), and they were also asked to express themselves in simulated social interactions online by posting and replying to sample comments using comment boxes and a threaded discussion forum (semidirected).

**Table 1:** Nine think-aloud usability social media task directives.

Task number	Task identifier	Task request
1	Create account	First, please create a log-in that will allow you to sign in to the website. Since you don’t already have an account, you will have to create one. Please complete *only* the fields that have an asterisk, and then click the sign-up button at the bottom of the page to create your account.
2	Review video labels	Next, please review the video category labels on the left-hand side of the computer screen in blue. Are there any category titles that you do not understand?
3	Rate video labels	Now, of these categories you just reviewed, please indicate which look interesting to you.
4	Locate video	Please find the *Deep Breathing* video under the blue *Stress Management* category label. Then click on the picture of the video and click the play button to view the video.
5	Post comment on video	Now that you have watched the *Deep Breathing* video, please post a one-sentence comment underneath the video indicating whether you found it informative.
6	Post/respond to discussion board	Next, please find the blue *Talk to Other People with COPD* category label at the bottom of the page, and post a one-sentence comment on one of the topics that are listed. Make the post about anything you feel like sharing. That’s the *Talk to Other People with COPD* category that we are asking you to locate.
7	Locate and play a recommended video	Now, click on one of the videos that are *Recommended for You*, and play the video. Please stop the video after 10 seconds.
8	Explore website^a^	Imagine you have just found this website online at your home or at the library. For the next 5 minutes or so, please explore the website however you would like. Feel free to talk about what you find, and tell us whether the health information you find is useful to you. This time is yours, so please use the website however you would like, and remember to tell us about your experience as often as you would like.
9	Sign out of website	For your final task, please sign out of the website.

^a^The think-aloud moderator was instructed to limit further exploration of additional website prototype functions after 5 minutes had elapsed. However, participation was not halted if participants were in the middle of watching a patient education video or actively contributing to a discussion thread. Participants who chose to stop exploring the website prototype before 5 minutes had elapsed were given the freedom to do so.

The moderator used modified usability methods [55] to motivate participants to talk out loud as they executed each task on a laptop computer. This modified methodology enabled the researchers to gain a better understanding of the cognitive processes that patients used to search for, and judge, the videos and functions of the social media website prototype. If the patient requested assistance during one of the tasks, the moderator instructed him/her to think about, and talk out loud about, alternative strategies. Mistakes were not addressed by the moderator, but were noted by two members of the research team who were taking field notes. If a patient requested assistance while thinking aloud, the moderator would ask participants to (1) make an attempt to repeat the task again, and (2) think again about what they were being asked to do. Assistance was provided if patients (1) chose not to explore an unfamiliar function independently (eg, posting comments to an online discussion board), (2) “froze” in front of the screen, or (3) verbalized that they were about to give up on the task [56]. If giving up was not explicitly verbalized, but the patient’s demeanor indicated confusion or frustration, then the moderator asked whether or not additional help was needed to continue. If the patient responded affirmatively with a verbal “yes” or a nod of the head, then assistance was provided.

Two methods were used to determine the reliability of the computer-based think-aloud usability test results: (1) member checking by the moderator and (2) cross-validation of paper-and-pencil field notes of participants’ experiences and comments documented by two members of the research team. Two researchers independently reviewed all transcripts and questionnaire data to control for systematic and response bias.

#### Phase III: Retrospective One-on-One Interviews (quant + QUAL)

Immediately following completion of the computer-based, think-aloud usability sessions, one member of the research team administered a series of structured usability and acceptability questionnaires with questions on demographics and use of electronic devices and the Internet to access health information. These structured surveys were followed by one-on-one, semistructured interviews where patients were asked to discuss their satisfaction with all website prototype features, including whether or not they found the social media features to be functional.

### Measures

#### Phase I: Evaluation of Design and Features of Social Media Website Prototype (qual)

Following each interviewee’s review of the mock-up, one member of the research team scheduled a telephone interview to ask five general semistructured questions regarding the design of a multimedia COPD patient education website (see Multimedia Appendix 1). Up to five tailored probing questions were also developed to request further clarification on responses pertaining to each interviewee’s area of expertise. Interviewees were also asked to discuss what types of interactive communication technologies would be appropriate for disseminating respiratory therapy education to medically underserved patients with COPD. All telephone interviews were audiotaped and responses were transcribed and reviewed.

#### Phase II: Computer-Based Think-Aloud Usability Session (QUANT)

While patients completed nine social media tasks using the social media website prototype, several measures were recorded, including task completion (*independent*, *with prompts* when intervention by the moderator was needed, or *incomplete*), task performance (*good*, *reasonable*, or *poor*), amount of time spent completing each task (both for independent completers and completers requiring moderator prompts), and the number of participant requests for assistance per task. Task performance was rated as *good* when operational skills were judged as adequate by two members of the research team, *reasonable* if the task was completed but not rated as *good* by both researchers, and *poor* if patients exhibited any difficulty when attempting the task. The following scale was used to evaluate level of agreement between researcher ratings of task completion, performance, and number of requests for assistance per task: 0 (*no agreement*), 1 (*a little agreement*), 2 (*much agreement*), and 3 (*total agreement*). Cross-validation of coder ratings across each category yielded intercoder agreement ranging between 2 and 3 (mean 2.8, SD 0.3). Differences in the distribution of codes were discussed by the two researchers during analysis until consensus was reached. In cases where consensus could not be agreed upon, a third researcher was consulted to reach a conclusion on all outcomes of interest.

#### Phase III: One-on-One Retrospective Usability Interviews (quant + QUAL)

Following each computer-based think-aloud usability session, patients were asked to complete a validated 18-item usability and acceptability questionnaire [57] to evaluate ease of use, usefulness, and satisfaction with the website prototype. Statements were evaluated on a 5-point Likert scale ranging from 1 (*strongly disagree*) to 5 (*strongly agree*). Data collected in this study using the 18-item questionnaire showed evidence of good internal consistency (Cronbach alpha=.89). Participants were also asked to answer demographic items on gender, age, education, self-reported COPD severity (*mild*, *moderate*, *severe*, *very severe*), experience using the Internet for health information, and eHealth literacy. The item eHealth literacy was measured using the eHealth literacy scale (eHEALS), a self-report instrument where participants are asked to rate eight statements related to comfort and skill finding, evaluating, and applying health information from the Internet on a 5-point Likert scale ranging from 1 (*strongly disagree*) to 5 (*strongly agree*) [58]. Data collected in this study using the eHEALS showed evidence of good internal consistency (Cronbach alpha=.83). Patients were also asked why other patients with COPD may or may not choose to use a fully functional, online version of the social media website prototype. Multimedia Appendix 2 lists the 10 open-ended questions—with associated probes—that participants were asked to consider.

### Data Analysis

#### Phase I: Evaluation of Design and Features of Social Media Website Prototype (qual)

Qualitative data from interviews with health care communication experts and patients with COPD were analyzed using ATLAS.ti version 7 qualitative analysis software. Thematic analysis [59] was used to identify overarching themes. One member of the research team transcribed the data and assigned brief verbal descriptions (ie, codes) to small chunks of data. Codes were altered and modified during the analysis based on the full picture of the data as ideas developed. On the basis of these emergent codes, themes from these interviews were identified to generate overall recommendations for the social media website prototype design.

#### Phase II: Computer-Based Usability Testing With Patients (QUANT)

The computer-based think-aloud usability sessions (eg, mouse movements, text inputs, navigation, verbalizations, video output, and cursor movement) were recorded using Camtasia Relay [60]. The level of assistance, performance, total amount of time to complete each task, and number of patient requests for help were entered into an SPSS version 21.0 database for analysis. Frequency and descriptive statistics were computed for all quantitative variables.

#### Phase III: One-on-One Retrospective Usability Interviews (quant + QUAL)

Patients’ sociodemographics, health characteristics, health-related Internet use, eHealth literacy, and scores on the 18-item website usability and acceptability questionnaire were entered into an SPSS version 21.0 database for analysis. Frequency and descriptive statistics were computed for all quantitative variables. A one-sample *t* test was conducted on usability and acceptability scores to evaluate whether the mean was significantly different from the midpoint (ie, 3 or *moderately agree*) of the 5-point response scale.

ATLAS.ti version 7 qualitative analysis software was used to code, label, and analyze all qualitative data collected from patients during the one-on-one retrospective interviews. Deductive constant comparison analysis [61] was used to identify chunks of data (ie, related portions of the transcripts) and group them into meaningful parts that were fit into codes established top-down according to four human-computer interaction categories from the literature [21,48,62]: (1) interaction and navigation (ie, the way users work with the site), (2) information architecture (ie, organization of links and hierarchy of content categories), (3) presentation design (ie, graphical interface and all visual elements of the page), and (4) information design (ie, preparation of communication products to achieve specified performance objectives). For example, the *interaction and navigation* category was subdivided into categories such as *user input*, *search options*, *loading speed*, *ability to click through buttons and icons*, and *help options*. Table 2 lists subcategories used to derive codes for each human-computer interaction category. When chunks of qualitative data did not fit codes within each of the four human-computer interaction categories, inductive codes were constructed and similar chunks were assigned to emergent themes. To calculate the frequency of themes identified during these interviews, a classical content analysis procedure [63] was used to count the number of times codes were applied to data.

**Table 2:** Subcategories referenced to derive top-down codes applied to qualitative transcripts.

Heuristic category	Subcategories applied to transcripts
Interaction and navigation	Consistency of features Able to click through buttons and icons Easy to scroll Visual and textual feedback based on user input Search options Loading speed Help options
Information architecture	Video placement and labeling Organization of topics and labeling Page design to facilitate task completion and intuitive access to content Dividing and classifying content into categories (ie, related topics grouped together) Descriptive labels with keywords that are easy to understand
Presentation design	Visual elements: form, content, arrangement, light (or contrast), and color Type size and legibility (font size, type of font, etc), layout, and visual searching Easy to read Text and background contrast Adequate white space Appropriateness of images Availability of external links
Information design	Ease of locating information Comprehensiveness Accurate, reliable, and credible health information Relevance of health information Empowering Willingness to return to the site General appreciation for the site Recommend the site to others in the future

## Results

### Phase I: Evaluation of Design and Features of Social Media Website Prototype

#### Patient Feedback

Patient interviewees indicated that they were pleased with the design of the preliminary website prototype (Figure 1), noting that use of a single website for health information on COPD would be both valuable and convenient to use. Furthermore, they appreciated the idea of being able to access an online clearinghouse of videos organized using clear links and picture-based screenshots. They also appreciated being able to communicate with other patients like them. To eliminate potential for confusion, patients also discussed the importance of using terminology that could be clearly understood by patients. One interviewee noted the need for videos that explained how COPD might feel on a day-to-day basis (eg, when to expect breathlessness, how to avoid stress, how to clean inhaler). Rigorous evaluation of the website before, during, and after development was also noted as important by the patient interviewees. For example, the URL planned for the website was originally “patientflix.com”, yet 4 of the 5 patient panelists suggested changing to domain name to include “COPD” within the URL to enable patients to locate the site using terms such as “COPD” on search engines like Google.

#### Expert Feedback

The use of structured social networking among patients was something viewed as valuable among the expert interviewees. One expert with experience working in pulmonary rehabilitation noted, “The more you can have information come from other patients, the better...they can get a lot out of talking to other patients.” Another medical doctor with expertise in pulmonary medicine supported the design of an on-demand, evidence-based video-sharing website to empower patients to answer personal disease-related questions. Of the expert interviewees, 3 with expertise in health communication research methods suggested keeping the scope of an online program small and simple to allow formative user feedback to drive further development of the site. These experts also suggested tracking engagement metrics to determine which patient education videos were most popular among users. Formative testing of the website with patients was viewed as important prior to releasing it online in order to determine how patients might want to access, use, and navigate the site to obtain and share health information.

### Phase II: Computer-Based Think-Aloud Usability Session

#### Patients’ Demographic Information

There were 8 patients with moderate (4/8, 50%) to severe (3/8, 38%) COPD who participated in the computer-based usability and acceptability testing. The patient group was made up of 5 (63%) males and 3 (38%) females. Patients ranged in age from 47 to 66 years old (median 54.5 years, interquartile range [IQR] 41.5-67.5 years), with both Caucasians (5/8, 63%) and African Americans (3/8, 38%) participating in the study. Patients reported living with several comorbidities, such as high blood pressure (6/8, 75%) and arthritis/joint problems (6/8, 75%). Most were either never married (3/8, 38%) or divorced/separated (3/8, 38%), and all patients reported completing high school (5/8, 63%) or possessing between 1 and 3 years of college-level education (3/8, 38%).

#### Computer-Based Think-Aloud Usability Session

##### Overview

The total length of time of the computer-based usability sessions ranged from 30 minutes, 24 seconds to 49 minutes, 26 seconds (median 36 minutes, 47 seconds), and the total completion rate of all 9 social media tasks attempted by the 8 participants was 93% (67/72)—58% independently (41/72) and 37% with moderator prompts (26/72). Table 3 describes completion times, performance, and time needed to complete each task during the think-aloud sessions.

**Table 3:** Completion, performance, time needed, and number of requests for assistance during each social media task among think-aloud participants (n=8).

Completion characteristic		Social media tasks									
		Task 1: Create account	Task 2: Review video labels	Task 3: Rate video labels	Task 4: Locate video	Task 5: Post comment to discussion board	Task 6: Post/ respond to discussion board		Task 7: Locate and play recommended video	Task 8: Explore website	Task 9: Sign out of website
**Level of assistance, n (%)**											
	Independent	3 (38)	8 (100)	8 (100)	4 (50)	4 (50)	1 (13)		4 (50)	5 (63)	4 (50)
	Moderator prompt assistance	5 (63)	0 (0)	0 (0)	3 (38)	4 (50)	5 (63)		3 (38)	3 (38)	3 (38)
	Incomplete	0 (0)	0 (0)	0 (0)	1 (13)	0 (0)	2 (25)		1 (13)	0 (0)	1 (13)
**Performance, n (%)**											
	Good	3 (38)	8 (100)	8 (100)	2 (25)	3 (38)	1 (13)		4 (50)	5 (63)	4 (50)
	Reasonable	3 (38)	0 (0)	0 (0)	5 (63)	5 (63)	2 (25)		3 (38)	2 (25)	3 (38)
	Poor	2 (25)	0 (0)	0 (0)	1 (13)	0 (0)	5 (63)		1 (13)	1 (13)	1 (13)
**Time for independent completion, minutes:seconds**											
	Median	2:16	0:21	0:28	0:14	0:28	1:03	0:27		4:22	0:22
	Minimum	0:46	0:08	0:15	0:09	0:54	1:03	0:22		3:03	0:05
	Maximum	3:53	0:52	1:19	0:37	1:22	1:03	0:35		7:23	0:40
**Time for completion with prompts, minutes:seconds**											
	Median	3:49	N/A^a^	N/A^a^	0:40	0:50	2:40	0:50		5:14	0:23
	Minimum	3:00	N/A^a^	N/A^a^	0:37	0:25	0:59	0:21		4:58	0:09
	Maximum	5:58	N/A^a^	N/A^a^	1:20	1:16	3:22	1:30		5:19	0:29
Requests for assistance made by participants, n		27	1	0	9	7	24	6		8	7

^a^Not applicable due to successful task completion by participants without prompts from the moderator.

##### Task 1: Create Account

Overall, patients struggled when attempting to create a personal log-in on the social media website prototype. Patients requested assistance from the moderator 27 times during the computer-based testing sessions. Only 3 of the 8 participants (38%) were able to complete this task independently. Creating the log-in also took longer than the majority of the other tasks, especially among participants who completed the task after being prompted to do so by the moderator (median 3 minutes, 49 seconds).

##### Tasks 2 and 3: Review and Rate Video Labels

All participants (8/8, 100%) were able to independently locate, read, understand, and comment on the importance of each patient education category label organized on the structured social media platform. Only 1 request for assistance was made during Task 2, and 0 requests for assistance were made during Task 3. Participants found the *Physical Activity*, *Infection Control*, *Medication Management*, and *Lifestyle* category labels to be among the most interesting.

##### Tasks 4 and 5: Locate and Post a Comment on Video

The majority of patients performed at least reasonably well when locating the *Deep Breathing* video within the blue *Stress Management* category tab (Task 4) and posting a sentence comment about whether or not it was informative (Task 5).

##### Task 6: Post and Respond to Discussion Board

Although the majority of patients were able to locate videos and post comments on them, they struggled to use a separate online discussion board embedded within the social media website prototype. Of the 8 participants, 5 (63%) performed poorly on this task, requesting additional prompts to access and add content to the discussion board threads. Of the 8 participants, 2 (25%) were unable to contribute to the discussion board at all, even after prompts from the moderator. Overall, participants requested assistance 24 times when attempting to complete this task.

##### Task 7: Locate and Play a Recommended Video

Half of the participants (4/8, 50%) independently located videos marked as *Recommended*. The other half of participants exhibited some difficulty deciphering which videos were marked as *Recommended* for them on the user interface. Overall, patients requested assistance during this task a total of 6 times, with half (4/8, 50%) of the patients recording a performance of *good*.

##### Task 8: Explore Website

Of the 8 participants, 5 (63%) spent between 3 minutes, 3 seconds and 7 minutes, 23 seconds (median 4 minutes, 22 seconds) exploring the social media website prototype independently. Participants receiving prompts from the moderator while exploring the website prototype spent more time using prototype functions (median 5 minutes, 14 seconds) when compared to participants who browsed the website prototype independently (median 4 minutes, 22 seconds). However, the variation in exploration time was less among participants who received moderator prompts (median range 4 minutes, 58 seconds to 5 minutes, 19 seconds).

##### Task 9: Sign Out of the Website

Participants exhibited difficulty determining how to sign out of the social media website prototype—only half (4/8, 50%) of the participants were able to sign out on their own.

### Phase III: One-on-One Retrospective Usability Interviews (quant + QUAL)

#### Patient Use of Internet for Health Information (quant)

Table 4 describes patients’ self-reported use of the Internet and social media/networking for health information. Patients reported accessing the Internet using different devices such as desktop computers (4/8, 50%), laptop computers (2/8, 25%), mobile phones (3/8, 38%), and other mobile devices such as iPads or tablets (2/8, 25%). Less than half (3/8, 38%) of the study participants reported using popular social media websites such as Facebook and Twitter for health information, and no participants reported belonging to online support groups or making entries into online health diaries/blogs.

**Table 4:** Self-reported Internet use for health information among think-aloud participants (n=8).

Method used to access health information		n (%)	
**Electronic device(s) used to access Internet** ^a^			
	Desktop computer	4 (50)	
	Laptop computer	2 (25)	
	Mobile phone	3 (38)	
	Mobile handheld device	2 (25)	
**Use of social media/networking for health information**			
	Facebook, Twitter, LinkedIn	3 (38)	
	Online support group	0 (0)	
	Online diary or blog	0 (0)	

^a^Participants could self-report using more than one device to access the Internet for health information.

**Figure 2:**
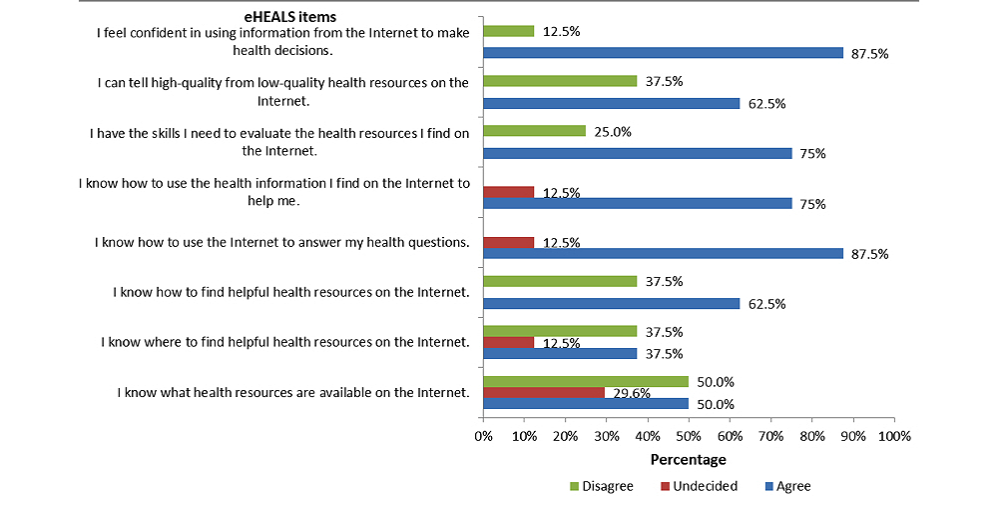
Participant responses to the eHEALS items (n=8).

Figure 2 illustrates the response frequencies of each eHEALS item. Responses were grouped into the following categories: *agree* (ie, *strongly agree* and *agree* responses), *undecided*, or *disagree* (ie, *strongly disagree* and *disagree* responses). The two statements with the highest level of agreement were, “I feel confident in using information from the Internet to make health decisions” (7/8, 88%) and “I know how to use the Internet to answer my health questions” (7/8, 88%). Statements with the greatest level of disagreement were related to knowledge of health resources that are available on the Internet (4/8, 50) and where to find helpful resources on the Internet (3/8, 38%).

#### Website Acceptability and Usability (quant)

As shown in Table 5, responses from the 18-item website usability and acceptability questionnaire ranged from 4 to 5 (mean 4.72, SD 0.33) on the 5-point Likert scale. The mean score on the questionnaire was significantly higher than the midpoint (3, *moderately agree*) of the rating scale (95% CI 4.48-4.96, *t*_7_=14.71, *P*<.001). Results from this survey indicated that participants were very satisfied with the purpose, layout, and functionality of the social media website prototype.

**Table 5:** Scores on the 18-item website acceptability and usability questionnaire among think-aloud participants (n=8).

Acceptability and usability item	Mean (SD)^a^
The website was easy to use.	4.88 (0.35)
I would recommend this website to others.	4.75 (0.71)
The information on this website was easy for me to understand.	4.75 (0.71)
The website made me think about something new.	4.13 (0.99)
The website was attractive.	4.25 (0.89)
The website was interesting.	4.75 (0.46)
I could tell the program was designed for patients with COPD like me.	5.00 (0.00)
I enjoyed using the website.	4.88 (0.35)
The website was useful.	4.75 (0.46)
The website could help me improve my COPD self-management skills.	4.50 (0.76)
The website was easy to navigate.	4.50 (0.76)
The graphics on the website went along with the information and videos that were presented.	4.75 (0.46)
I liked the colors used on the website.	4.75 (0.71)
I liked the font that was used on the website.	4.88 (0.35)
I liked the way the website was organized.	4.63 (0.52)
I liked the way the website screen layout looked.	4.75 (0.46)
I thought the information presented on the website was relevant to me.	5.00 (0.00)
I would use the website the next time I am looking for COPD self-management information.	5.00 (0.00)

^a^Scale responses ranged from 1 (*strongly disagree*) to 5 (*strongly agree*).

#### Usability Strengths (QUAL)

##### Overview

Most patients (6/8, 75%) noted that using most of the social media functions on the website prototype was easier than expected. One patient stated the following:

I was actually impressed, because I was a little nervous (at first). When I first got in front of the computer, I was like “Oh God”, but the way it (the website prototype) was set up, it wasn’t scary. If I can do this, a lot of other people can

Patient 1, male, 53 years

Several patients described the clickable tabs as “empowering” by enabling them to self-select videos and materials applicable to their own disease-related concerns. One patient noted the following:

See, I don’t smoke so that isn’t a big interest to me (cursor pointing to smoking cessation), but for other people it could be.

Patient 8, female, 55 years

Another patient commented the following:

See like now, with the list about what else I can do, I can experiment with that. You know what I’m saying. And it will educate me more than I have going on now.

Patient 7, male, 49 years

##### Reaction to Role Model Actors

Of the 8 participants, 3 (38%) responded positively to the clickable video boxes containing screenshots of age-appropriate, diverse role model actors. One participant commented on benefitting from the following:

...what they are showing you about what you want to do and then they (video narrator) would explain it with the vocal part too.

Patient 4, male, 64 years

Another participant commented the following:

Plus, they are showing real-life people doing what they are supposed to be doing. People can read stuff, but when they actually see a video—this is how you use this medicine, use as the doctor says, or use as directed, you got a video showing how to (actually) use it properly...you’ll get the maximum effects.

Patient 3, male, 54 years

##### Likelihood to Visit Website

All participants (8/8, 100%) strongly agreed that they would use the website the next time they were in need of information on COPD, especially if they were at home. One participant noted the possibility of visiting the site using a mobile device (eg, mobile phone, tablet computer), stating the following:

*If I was at work and having problems, I would go through it. I would use it at home, because if you have an iPad, you can pull it up. You know your phone, because you never know if you are going to be out somewhere and have problems, you can go, “Oh, let me look over here.”* [Patient 8, female, 55 years].

#### Usability Violations (QUAL)

##### Overview

While very positive feedback was obtained during the follow-up retrospective usability interviews, there were also several usability violations that were identified. Below is a synthesis of perceived usability violations discussed according to two heuristic categories assessing human-computer interaction—*information design* and *interaction and navigation*.

##### Information Design

The majority of participants reported difficulty locating links to sign in and log out of the website prototype. Participants described difficulty signing in and out of the website prototype 19 times—it was the social media website prototype’s most severe usability violation in this human-computer interaction category (Table 6). Several participants expected a sign-out or log-out button to be positioned at the bottom right-hand corner of the page, yet, in the website prototype that was evaluated by patients, the log-out link was placed in the upper right-hand corner, causing participants to experience some confusion. Half of the participants (4/8, 50%) also reported some difficulty locating patient education videos under different self-management categories. Comments related to this violation were coded 9 times, with participants noting that some video heading labels were vague and needed more clarification (Table 6).

**Table 6:** Two main heuristic categories with usability violations.

Heuristic category	Usability violation theme	Frequency of codes^a^, n
**Information design**		
	Difficulty locating sign-in and log-out links on user interface.	19
	Confusion locating videos in certain self-management categories.	9
**Interaction and navigation**		
	Frustration with using discussion forum applications.	46
	Difficulty filling in fields and submitting user information using the log-in interface.	19
	Confusion navigating to self-management category tabs that contained sought-after videos.	9

^a^Identified within themes during constant comparison analysis.

##### Interaction and Navigation

Of the 8 participants, 7 (88%) discussed having difficulties using the group discussion forum. Comments related to this difficulty appeared 46 times in the transcript (Table 6). Of the 8 participants, 5 (63%) participants specifically commented that the topic-delimited threaded discussion board was disorienting and confusing. Participants described feeling reliant on the moderator to “tell them what to click on” in order to enter and reply to comments. In addition, 4 of the 8 participants (50%) discussed frustration when entering information into fields for creating a unique user account. Several participants also noted that they were reluctant to seek out videos when additional scrolling was needed to navigate to video category labels. Of the 8 participants, 2 (25%) attributed their difficulty with navigation to visual problems experienced when looking at the laptop screen. One participant noted the following:

I just got to keep the screen close to me for my vision. These glasses are going out. My sight changes with my diabetes.

Patient 5, male, 66 years

## Discussion

### Principal Findings

Usability evaluation is a useful and cost-efficient way to formatively test patient education websites [5,33,64]. In this study, a multidisciplinary group of health care communication experts and COPD patients collaborated to develop and evaluate a low-computer-literate social media resource center focused on COPD patient education. Patients in this study reported higher than anticipated education levels (ie, all had graduated from high school) and also self-reported the use of a variety of electronic devices to access the Internet for health information. Overall, patients in this study reported feeling confident using information from the Internet to make health decisions and answer health-related questions, yet fewer were confident in their ability to locate health information on the Internet. A little less than half of the participants reported current use of social media to find and share health information.

### Usability Strengths Present in Social Media Website Prototype

Overall, the majority of the violations identified during a heuristic evaluation of the website prototype [36], conducted prior to patient-based usability testing, were not discovered in the computer-based think-aloud testing reported here. This was not unexpected, because most usability problems identified via heuristic evaluation are minor, very specific, and cause little trouble for the system’s users [65]. All participants in the usability study strongly agreed that the website prototype was easy to navigate and designed for patients like them. While questions remain regarding the effects of educational components in COPD self-management interventions and respiratory therapy programs [66], evidence suggests that effective communication between patients with COPD and their providers is associated with better quality of care and confidence in dealing with breathing problems [67]. There is a need for further exploration into innovative methods for using social media to support more effective patient-provider communication. Use of social media and mobile devices such as mobile phones and tablet computers may improve accessibility to COPD patient education. This would fill a key gap inhibiting effective patient-provider communication related to the prevention and management of breathing exacerbations.

Almost 60% of adults in the United States and 61% of people worldwide own a mobile phone, while over 40% of Americans and 17% of individuals globally own a tablet computer [68,69,70]. Patient use of mobile devices increases the potential to disseminate patient education to individuals who demonstrate at least moderate levels of eHealth literacy, and have the means to purchase and benefit from mobile devices connected to the Internet. However, as mobile medical apps continue to show much promise for delivering a range of patient interventions, including patient education, it will become important to monitor and manage the quality and safety of using mobile devices for delivering health information to patients [71]. Moreover, it is suggested that comprehensive usability testing be conducted on chronic disease self-management prototypes that are designed for use on mobile platforms.

### Usability Weaknesses Present in Social Media Website Prototype

Similar to other usability research evaluating health-related websites [33], users had difficulty signing in and logging out of their user accounts on the website prototype. Despite this difficulty, most participants discussed how having a personal log-in account on the social media site would help them feel as if they officially belonged to a patient community. Also, participants were very dissatisfied with the interactive threaded discussion board. They disliked having to locate a self-management discussion topic link from among 10 possible options, demonstrating difficulty determining which links needed to be clicked to post an original comment and/or reply to comments made by others. Most participants remarked that submitting a post to comment boxes placed underneath discrete videos represented a far less cumbersome task.

### Social Media Website Prototype Redesign and Modification

Mixed-methods data from the computer-based think-aloud session and retrospective one-on-one interviews informed the development of a system redesign plan that focused on making improvements related to two primary usability heuristics—*information design* and *interaction and navigation*. In total, 13 changes were identified and proposed for the social media website prototype categorized into the information design (n=7) and interaction and navigation (n=6) heuristic categories (Table 7). Modifications took approximately 4 months to complete.

**Table 7:** Website prototype design resolutions made following usability testing with participants.

Heuristic category	Specific examples of design resolutions
Information design	Increased visibility of the sign-in link by embedding it into the *Welcome* tab in large, bold sans serif font. Modified screenshots of videos that were not reflective of video content. Split up 15-minute video on *Diet and Nutrition* into 11 shorter video segments. Added a video dictionary of COPD terms (A-Z) for users to reference when encountering unfamiliar terms. Edited user comment tool to make it less disorienting when uploading and responding to comments. Complete redesign of discussion board. Removal of asp.net MVC Open forum discussion platform to be replaced with more user-friendly interactive forum technology.
Interaction and navigation	Increased font size requirements for username label, written comments, and reply links within discussion forum. Incorporated Google Analytics into back end of the website prototype to track number of activated sessions, average session duration, bounce rate, number of page views per session, percentage of returning and new visitors to the site, time spent on the site, etc. Decreased number of required fields to become a registered user on the site. Made clear headings with arrows on page to direct users to click on recommended videos. Made all video labels describing content clickable (originally only the screenshot was clickable). Improved site architecture to limit the need for scrolling to locate online videos and resources.

General examples of technical improvements included reprogramming some video content, modifying and sharpening the design of the user interface, and improving the interactive functions to make them more intuitive. Other more specific examples included (1) improving the scroll-bar function to limit the need for scrolling to find videos and post/reply to user comments, (2) modifying the log-in tool to reduce the number of required fields by half, and (3) changing the location of the sign-in link on the home page by placing it in a more prominent location on the *Welcome* tab. The computer software used to design the discussion forum was also supplanted with a more user-friendly interactive technology to make the discussion board easier to use (with fewer customization options). We expect these changes will facilitate more meaningful use among patients and informal caregivers, with fewer possibilities for errors when posting and replying to comments from fellow users.

**Figure 3:**
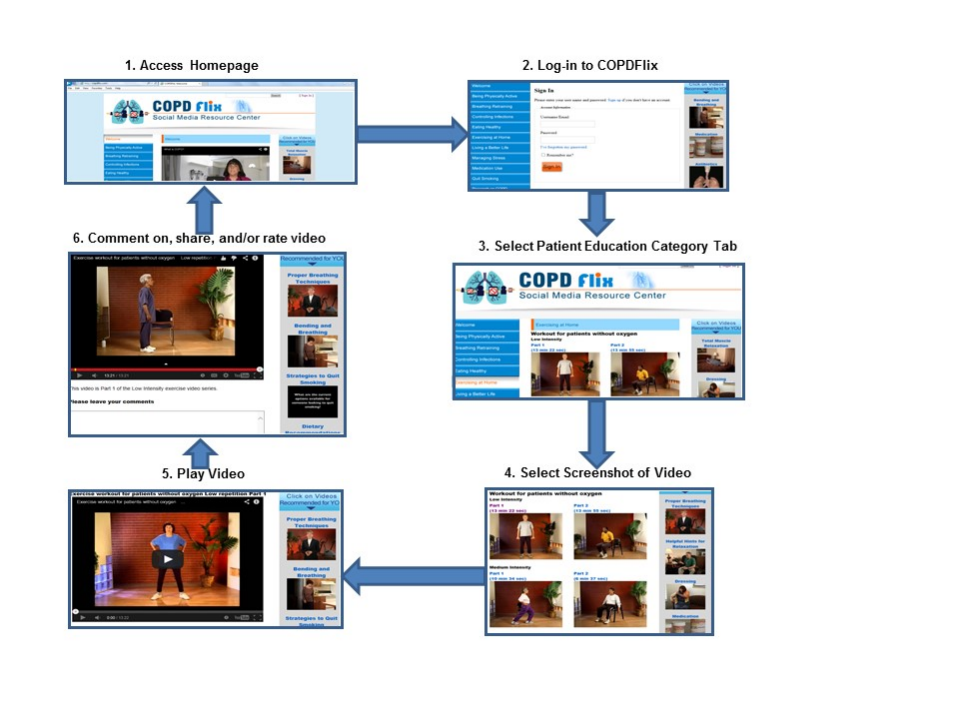
Screenshots of beta version of COPDFlix social media resource center, modified following usability evaluation.

In addition, several visual presentation elements were refined to create a more ergonomically functional design. For example, the tab containing videos recommended for specific users (ie, *Videos Recommended for You*) was modified to make the tab heading and video labels more distinguishable by increasing the font size, putting “YOU” in all capital letters, and using dark blue text (instead of orange text) to provide a better contrast with the light blue background. The research team also completed a thorough review of all hyperlink labels and changed 18 category label headings to make terms more understandable and action oriented (ie, beginning with verbs). Brief two-to-three-sentence written descriptions of each video were also placed underneath each video screen to clearly explain the content covered in each video (Figure 3).

### Study Limitations

Although usability evaluation examines the relationship between users, technological tools, and associated tasks in a specific working environment, user performance and acceptance in a controlled setting does not always convey the entire picture regarding usability. Even though describing system features, functions, and interfaces from the user perspective is essential to ensure the design of usable systems, perspectives from a limited sample of potential users may not be generalizable to larger patient populations [72,73]. Small-scale usability testing is also not sufficient for generating metrics that can be analyzed through common statistical tests [74]. It is important to note, however, that a small sample of approximately 8 subjects is the minimum recommended sample size for determining satisfaction and usability in formative testing of computer-based systems [52,75,76]. In addition, participants in this small sample included representation from females (3/8, 38%) and African Americans (3/8, 38%), two subpopulations often underrepresented in studies of individuals with COPD.

In the current study, the modified think-aloud protocol provided the moderator with the flexibility to intervene during the computer-based usability sessions to encourage participants to continue on with tasks, which may be considered a source of error leading to distorted self-reports [55]. The modified think-aloud technique can redirect participants’ attention to a self-evaluation of task accomplishment, which could change their thought processes. However, in this study of medically underserved patients with COPD, participants seemed to value additional prompts from the moderator to help point them in the right direction. To decrease the potential for experimenter bias, the moderator was instructed to minimize intervention during the verbalization process by only reminding each subject to “keep talking” if and when subjects stopped talking in front of the computer. This subtle intervention generally does not disturb and/or bias ongoing cognitive processes among users [55]. However, there were times during the think-aloud protocol when participants stopped thinking out loud when they were concentrating on a given task, and instead started talking about their personal health issues, especially when they saw topics on the social media website prototype that coincided with their personal health issues. Retrospective questionnaires and interviews administered following the computer-based think-aloud protocol likely minimized the effects of any cognitive or task flow interruptions experienced by patients [21,52].

Patients in this usability study self-reported higher than expected education levels and moderate eHealth literacy, which limits the applicability of results to older patients with COPD who may possess lower education levels and minimal experience using the Internet. In this study, we were unable to evaluate environmental factors (eg, Internet access points, connection speeds) that may influence use of a social media resource center among diverse patient populations. Patients were also only exposed to the social media website prototype at one point in time, limiting our ability to examine any longitudinal effects of visiting various sections of the site at variable doses over time. Evaluating new technology with iterative rounds of testing is a key principle of prototyping, so the lack of a second round of computer-based usability testing precluded the collection of additional feedback regarding the revised social media website prototype. Future pragmatic clinical trials should attempt to further evaluate the usability, acceptability, exposure, and engagement with the subsequent Web-release version of the prototype among larger cohorts of patients with COPD.

### Conclusions

Human-computer interaction researchers in health care have acknowledged the need for a comprehensive, integrated model for human-computer interaction with health care technologies [21,77]. In this study, mixed-methods stakeholder feedback was used to make design recommendations, categorize usability violations, and prioritize potential solutions for improving the usability of a social media resource center for COPD patient education. Combining conventional methods of computer laboratory testing with low-cost qualitative methodologies enabled our multidisciplinary research team to use qualitative and quantitative data to make targeted modifications to a COPD social media resource center website prototype, later named “COPDFlix” prior to Web release. The website prototype possessed moderate error rates, and was generally well-received and perceived to be learnable among a community-based group of medically underserved older adults with COPD. While we were unable to address every usability problem identified during testing, we were able to prioritize the most important problems in need of modification. Future studies should explore pedagogical methods for teaching patients how to use social media to locate and evaluate evidence-based health information on preventing and managing behavioral risk factors associated with chronic diseases like COPD. Integrating use of a social media resource center within comprehensive COPD patient education programs may help to improve patient outcomes, such as health-related quality of life, exacerbation frequency, and cost of care.

## Multimedia Appendix 1

Key informant interview rubric.

## Multimedia Appendix 2

Retrospective one-on-one interview questions on experience using the prototype (with probes).

## Abbreviations

CEnR: community-engaged research
COPD: chronic obstructive pulmonary disease
eHEALS: eHealth literacy scale
IQR: interquartile range
IRB: Institutional Review Board
WDMHC: Website Development Model for the Healthcare Consumer
