# Supplementary material for: Engaging Community Stakeholders to Evaluate the Design, Usability, and Acceptability of a Chronic Obstructive Pulmonary Disease Social Media Resource Center
Source: JMIR Res Protoc. 2015 Jan 28;4(1):e17. doi: 10.2196/resprot.3959 (PMC4336200; doi:10.2196/resprot.3959)
Supplement: Supplementary file 1 [file resprot_v4i1e17_app1.pdf]

Additional File 1. *Key Informant Interview Rubric*

General Question	Probing Questions
In general, what do you think of the idea to deliver COPD self-management education using Internet videos on COPD self-management education?	• *What potential issues do you see with this concept?*<br>• *What do you like about the idea (if anything)?*<br>• *What do you dislike?*<br>• *How can any potential problems be ameliorated?*
The concept to have patients "self-tailor" their education with the ability to self-select which videos and topics they'd like to watch is unique to COPD self-management education. What are your thoughts on the "self-tailoring" concept for this website?	• *From your experience, do you think this will enhance the usability and marketability of the website?*<br>o *If so, how?*<br>o *If not, why not?*
In reviewing the list of COPD self-management education topics to be included in *patientflix.com*, is there anything we are missing in terms of topics to cover?	• *What topics?*
In reviewing the mock diagram of how we envision the *copdflix.com* website to look, feel and be organized, what do you think of the overall structure and design of the website concept?	• *What aspects do you like?*<br>• *What aspects do you not like?*<br>• *Do you have suggestions for changes?*
How do you envision the website being introduced to COPD patients?	• *In what setting?*<br>• *By whom?*<br>• *Through what delivery system or mechanism?*
