# Supplementary material for: Engaging Community Stakeholders to Evaluate the Design, Usability, and Acceptability of a Chronic Obstructive Pulmonary Disease Social Media Resource Center
Source: JMIR Res Protoc. 2015 Jan 28;4(1):e17. doi: 10.2196/resprot.3959 (PMC4336200; doi:10.2196/resprot.3959)
Supplement: Supplementary file 2 [file resprot_v4i1e17_app2.pdf]

Additional File 2. *10 retrospective one-on-one interview questions on experience using the prototype (with probes)*

Question Number	Interview Question	Probes (when applicable)
1	In general, what did you think of the COPD website?	*Information, layout, videos, discussion board. How do you feel about the accuracy, trustworthiness, and usefulness of the information? What information was most helpful? Was it easy to understand?*
2	How would you describe the ease/difficulty of using the website?	*What challenges did you have while navigating through the website? What would make it easier to use?*
3	What aspects of the website did you like the best?	*Information, videos, ability to pick the videos you wanted to view, ability to leave comments, see recommended videos, etc. Can you tell me more about that?*
4	What aspects of the website did you like least?	*Information, videos, ability to click the videos you wanted to view, ability to leave comments, see recommended videos, etc. Can you tell me more about that?*
5	What did you think about the overall look of the website?	*Design, colors, images, layout, etc. Does the website feel warm and friendly or cold and technical? Can you elaborate on that? Is the program visually appealing? How could we improve the visual appeal?*
6	Would you use this website?	*If yes: How would you use it? When would you use it?*<br><br>*If no: Why not? What might prompt you to use the website? Why?*
7	What do you think are the best ways for getting the website out to other people with COPD?	
8	Do you think this website would be helpful to other people with COPD?	*Why or why not? How do you think other patients would use the website to learn about managing COPD? What would interest them most about the website?*
9	If you could make changes to the website, what changes would you make?	
10	Is there anything else that you would like to share with us on the website idea that you think would be helpful to us as we further develop the site?	
